# Creativity and its relationship with intelligence and reading skills in children: an exploratory study

**DOI:** 10.1186/s41155-022-00221-3

**Published:** 2022-06-10

**Authors:** Roberta Louise Mariano Bezerra, Rauni Jandé Roama Alves, Cíntia Alves Salgado Azoni

**Affiliations:** 1 Laboratório de Linguagem Escrita, Interdisciplinaridade e Aprendizagem (LEIA), Universidade Federal do Rio Grande do Norte (UFRN), Departamento de Fonoaudiologia. Centro de Ciências da Saúde. Rua Gen. Gustavo Cordeiro de Farias, s/n - Petrópolis, Natal, RN 59012-570 Brazil; 2 Laboratório de Neuropsicologia e Avaliação Psicológica (NeuropsiLab), Universidade Federal de Mato Grosso (UFMT), Av. Fernando Correa da Costa, 2.367 – Bairro: Boa Esperança, Cuiabá, MT 78060-900 Brazil

**Keywords:** Creativity, Intelligence, Phonological awareness, Decoding, Learning

## Abstract

Creativity, intelligence, and reading skills such as phonological awareness and decoding in reading can be critical to academic success, especially during childhood. Thus, this study aimed to characterize creativity, intelligence, phonological awareness, and reading decoding and verify possible relationships between creativity and these skills. The sample consisted of 75 children divided between the 1st, 2nd, and 3rd grades of municipal public schools in the Brazilian context. The results indicated the gradual evolution of creativity, intelligence, phonological awareness, and reading decoding in children from the 1st to the 3rd year, especially for the performance of the 3rd year. Correlations between creativity with intelligence and reading skills were also evidenced for all three classes, with the 3rd year with stronger correlations, which are promising results for these relationships. The study of creativity is still a recent field for empirical investigations and deserves future investigations for a better understanding of these constructs in this population.

## Introduction

Currently, the apprehension of formal knowledge occurs during the school experience, in which new experiences are shared, socio-emotional abilities are discovered, and cognitive skills are developed, such as verbal skills, reading, writing, and intelligence, among others. Creative ability is also part of this process and includes curiosity, fantasy, and imagination (Glaveanu, 2011). In addition, it can also promote good school performance (Fanchini et al., 2018).

Creativity can be considered a fundamental skill nowadays as a differential for success, quality of life, and mental health, especially after the global pandemic that started in 2020. In the daily routine, the investigation of this skill has been carried out from two directions, the first one that would be focused on the creativity of daily activities, such as problem solving at school and at work, activities with the arts, literature, cooking, that is, everyday creativity, called Little c (creativity); and a second, aimed at more robust and complex creative activities, of renowned personalities in society and individuals who stand out with great creative achievements, called Big C (creativity) (Kaufman & Beghetto, 2009; Kornilov et al., 2016).

In general, the determination of these performances can be understood in a multidimensional way, as they may be involved, in addition to cognitive skills, personality characteristics, and environmental influences (Runco, 1999). Following this thought, Torrance (1993) defined creativity as a multifaceted process that involves being aware of problems and gaps in knowledge, identifying omissions in information, making assumptions about these gaps, analyzing and testing these hypotheses, retesting and revising them again, and, finally, communicating their results and promoting changes in the environment. This perspective is in line with one of the most accepted theoretical and methodological models, with a multitrack and multimethod empirical basis, both for their understanding and for evaluation, there is the one known as “4P (person, press, process, product)” in which creativity can be investigated from these 4 variations (Rhodes, 1961; Lassig, 2020). And these aspects reinforce its investigation in different contexts, models to assess it and its relationship with other skills, as conceptually there would be all these factors involved in the determination of creativity (Nakano, 2020).

Among the skills that can mediate these performances, intelligence is related to the creative process. However, while being confirmed, this relationship is also questioned in the field of sciences (Krumm et al., 2014; Pan & Yu, 2016). The data have pointed to different levels of covariance, such as that they are disjunctive skills, that is, it does not take a high intelligence for a person to be creative and vice versa (Getzels & Csikszentmihalyi, 1972; Sternberg & O'Hara, 1999). Alternatively, the opposite is that they are strongly related and overlapping, in which creativity is necessary for intelligent behavior to manifest (Sternberg & O'Hara, 1999). There is also the hypothesis/theory of the threshold, in which creativity and intelligence would be related from a certain point, mainly from a certain level of intelligence. For example, there would be positive relationships between high creativity and intelligence based on the Intellectual Quotient (IQ) close to/equal to 120 (Karwowski & Gralewski, 2013; Shi et al., 2017).

Thus, more studies must be carried out in this area in order to clarify these relationships further. However, some limitations exist for this to happen, mainly in Brazilian territory and for the child population. There is only one validated and standardized instrument for this reality, the Test of Children’s Figural Creativity (TCFI), which assesses the creative process. Interestingly, among the hypotheses raised that would explain these discrepant correlations, there are those that such variations would result from the type of creativity and intelligence being evaluated, the differences between the instruments, the types of research, and the types of participants that compose the sample (Nakano, 2012; Pan & Yu, 2016).

The TCFI was developed and based on the Torrance Creative Thinking Test, which significantly influenced the assessment of creativity and the method for its measurement (Abdulla & Cramond, 2017). In an investigation by Alves and Nakano (2015), when verifying correlations between creativity and intelligence of children with dyslexia, the TCFI, the Wechsler Intelligence Scale for Children (WISC-III), and Raven’s Colorful Progressive Matrices (RCPM) were used. The results mostly pointed to the absence of significant correlations between intelligence and creativity. Likewise, in a research by Nakano and Brito (2013), low correlations were observed between the instruments through the TCFI and the Child Thinking Test Battery with elementary school children. In contrast, in an investigation by Nakano (2012), TCFI and Human Figure Drawing (HFD) were used, and moderate correlations between skills were found. In other words, there was no pattern in Brazil between the correlations, again reinforcing the need to deepen this issue.

From another perspective, creativity can also relate to other constructs, such as school performance and academic achievements in mathematics, writing, reading, and science, among others (Gajda et al., 2017; Leopold et al., 2019; Bart et al., 2020). Among these skills, reading can be highlighted, which allows practices such as critical thinking, reasoning, curiosity, and freedom of expression, characteristics commonly associated with creative behavior (Wang, 2012).

Torrance (1974), in a study with teachers, suggested using activities focused on creativity to facilitate the understanding of the knowledge of children with dyslexia through storytelling as a way to motivate them to foster critical, creative thinking and develop the reading process. Anderson and Gipe (1983), in an investigation focused on textual comprehension with elementary school students, observed that the students presented more adequate performances, and reading comprehension was the most creative children. Likewise, Wang (2012) and Saeed et al. (2013) also showed that students who had higher reading and writing speed achieved significantly better performance in creative performance.

That said, it appears that the literature has demonstrated relationships between creativity and reading, yet, among the different stages and processes involved in the act of reading, phonological awareness and reading decoding can be highlighted because they have greater relevance at the beginning of reading development (Lerner & Lonigan, 2016). However, in a short search in the PubMed database, no research was found on the relationships involving creativity with phonological awareness and reading decoding until the time of this study, early 2021. However, investigating possible associations between these skills becomes original, as it involves initial stages of development of the written language and consequently the effective development of reading.

Phonological awareness is responsible for reasoning about the sound system of language, competence to reflect on speech segments, and awareness of the decomposition of words into disparate components (Soto et al., 2019). It is also due to the quality of this ability that reading decoding is developed, which allows the identification of written codes, associating them with the meaning of what is exposed in the text and the good development of the decoding and recognition of words, in addition to enabling a better understanding of the stimulus read also facilitates the reading speed, with the increase in speed, the reading becomes adequate, fast, and fluent, and consequently, the accurate and satisfactory reading of the text occurs (Cunha et al., 2017; Santos et al., 2017). Furthermore, its assessment assists in diagnosing conditions such as the Specific Learning Disorder that affects reading, dyslexia (Santos et al., 2017). Given these gaps exposed above, the present study aimed to characterize creativity and verify its relationships with intelligence, phonological awareness, and reading decoding in Brazilian children belonging to the mandatory school initials in the country, specifically, from first to the third grade of Elementary I.

## Method

### Participants

The sample consisted of 75 children, of both sexes, from Elementary School I, from Natal/RN in the Brazilian context before the COVID-19 pandemic. The children were chosen randomly from the inclusion criteria: belonging from the 1st to the 3rd year of Elementary School I in the municipal public schools of Natal, and exclusion: hypothesis history or confirmed diagnosis of neurodevelopmental disorders and/or hearing, motor, and visual alterations and absence of response in more than half of the applied tests’ items. The students came from two municipal schools, divided respectively into 1st year: 23 children, with 51% (*n* = 12) being female, ages 6 to 7 years (M = 6.78; SD = .42); 2nd year: 26 children, being 50% (*n* = 13) female and aged between 7 and 8 years old (M = 7.69; SD = .47); and 3rd year: 26 children, with 46% (*n* = 12) female, aged between 8 and 9 years (M = 8.73; SD = .45). The Kruskal-Wallis test was used to compare ages (*χ*^2^ = 57.674, *p* <.001), and a statistical difference was found between the groups. The epsilon square was .77, indicating a solid effect size. The post hoc test (Dunn) also identified a difference when the groups were compared two by two. Gender was compared between classes using the chi-square test, which did not show statistically significant differences (*χ*^2^ = .184; *p* = .912). The size of the effect, assessed using Cramer’s V, indicated a small effect (.050).

### Materials

The following instruments were used to assess the skills of creativity, intelligence, phonological awareness, and reading decoding, subsequently:

- *Brazilian Figural Creativity Test (TCFI)*, an instrument developed by Nakano et al. (2011), aims to assess creativity through figures. It is published with evidence of validity and rules for the age group of the 1st to 8th grades (2nd to 9th grade) of Elementary School. It can be applied individually or collectively, with 25 min to perform the activities, and can be limited to 40 min in total, adding instruction time, delivery of materials, etc. It consists of 3 activities that must be answered in the form of drawings, with the assessment of creativity through a score on 12 creative characteristics: fluency, flexibility, elaboration, originality, expression of emotion, fantasy, movement, unusual perspective, internal perspective, use of context, the extension of the limits, and expressive titles. Such characteristics can be grouped into four factors which will be correlated with the factors/total scores of the rest of the instruments. The factors are (1) enrichment of ideas, which is about improvement, commitment, and dedication to ideas and good organizational and planning skills. Competence to see things from different perspectives by understanding the problem from a larger perspective; (2) emotionality, about emotional skills and persistence in your ideals with confidence without fear of criticism, ability to believe in ideas and plans without fear of failure; (3) creative preparation, competencies aimed at controlling impulsiveness, when faced with a problem, there is the analysis of response possibilities and the choice of the best solution before taking action; and (4) cognitive aspects, based on the characteristics of originality, flexibility, fluency, and extension of limits, this factor comprises aspects focused on innovation, redefinition of problems, openness to the new and rupture with prejudices, generation of multiple ideas, and characteristic of production of ideas that deviate from the conventional, traditional. Validity evidence was performed with the Torrance figures creativity test (Wechsler, 2002), to which evidence of concurrent validity between the two instruments with values between .81 and .94 was found. For accuracy, the test-retest method was used with results that showed values between .84 and .95 of correlation.
- *Wechsler Abbreviated Scale of Intelligence (WASI)*, adapted for the Brazilian territory by Trentini et al. (2014). WASI is an abbreviated intelligence scale adapted from the Wechsler Intelligence Scale with evidence of validity and Brazilian standards described in its manual for children from 6 years of age up to 89 years old. It is administered individually, with an average application time of 30 min. Composed of four subtests, vocabulary, cubes, similarities, and matrix reasoning, they evaluate verbal knowledge, visual information processing, spatial and non-verbal reasoning, and fluid and crystallized intelligence. The vocabulary and similarities subtests make up the verbal index that will generate the Verbal Intellectual Quotient (VIQ), responsible for approaching a measure of crystallized intelligence. The cubes and matrix reasoning subtests provide the execution index that will outline the Intellectual Execution Quotient (IEQ), responsible for approaching a fluid intelligence measure. In all, all subtests make up the Intellectual Quotient (IQ) of the full scale. Evidence of convergent validity was performed employing significant correlations (*p* <.05) of all WASI subtests with the subtests corresponding to the WISC-III scale, with correlation coefficients with values of .44 for vocabulary, .61 for similarities, and .65 for cubes. Significant statistical correlations were also found (*p* <.05) of the WASI substances with the corresponding subtests on the Wechsler Intelligence Scale for Adults, third edition (WAIS-III) (Nascimento, 2004) with values of .63 for vocabulary.
- *Phonological Awareness - Sequential Assessment Tool (CONFIAS)*, an instrument developed by Moojen et al. (2013), aims to assess phonological awareness. It can be used to evaluate the educational process of illiterate children, and in the process of literacy, its form of application is individual with an age range from 4 years and without age limit. The test consists of two parts; the first refers to syllabic awareness, formed by nine items: synthesis, segmentation, identification, production, exclusion, and syllabic transposition; the second part corresponds to the awareness of the phonemes, through seven items: word production that begins with the given sound, identification of the initial phoneme, identification of the final phoneme, exclusion, synthesis, segmentation, and transposition. Syllabic tasks add up to 40 points, and phoneme tasks add up to 30 points, accounting for 70 points in total.
- *Reading Assessment of Words and Pseudowords Isolated (LPI)*: the LPI was developed by Salles et al. (2017) to evaluate the performance in reading words and pseudowords of children and adolescents, from 6 to 12 years of age. Its application is individual, taking around 15 min. It aims to assess oral reading ability, with precision in recognizing words and pseudowords, and investigate reading strategies that are preserved and deficient by measuring qualitative and quantitative scores. The instrument has 59 stimuli, divided between 19 regular words, 20 irregular, and 20 pseudowords, matched by the frequency of the words and the extension (short and long). The general factors used in this study for interpretation and correlation with the other instruments were the raw total (sum of previous items) and the total percentile. Evidence of convergent validity was verified with NEUPSILIN-Inf (Salles et al., 2015) in word writing tasks, writing of pseudowords, and writing of words and pseudowords (total score) with values of .43, .31, and .46 (*p* <.05), respectively. There was evidence of convergent validity also between the word writing subtest of the School Performance Test - TDE (Stein, 1994) with the scores of the tasks of reading regular words and pseudowords of the LPI with correlations of values .76 and .61 (*p* <. 001), respectively, and strong correlation with the irregular word reading scores and total reading score in the LPI of .80 and .76 (*p* <.001).

### Procedure

Initially, the choice of municipal schools where the research would take place was made for convenience. Then, the study was submitted and approved by the Research Ethics Committee (CEP) of the Federal University of Rio Grande do Norte - UFRN (Protocol: CAAE N° 85593418.0.0000.5537). For the participants’ recruitment, a meeting was previously held with the parents/guardians on the scales to clarify the study, being requested to sign the informed consent form for those who authorized their children’s participation. After this procedure, the evaluations with the children were scheduled and also carried out in schools. Such participants were removed individually during the class period (except the break) to a room prepared with minimal acoustic conditions for evaluation. For the application of all instruments, it took approximately three 40-min meetings with each one. It is noteworthy that they were presented with the Term of Free and Informed Consent in the initial session. If they agree to participate in the research, they should sign it. All agreed, with no sample loss in this regard.

### Statistical analyses

For data analysis, descriptive statistics (mean, standard deviation, frequency) were performed for each instrument, considering the general sample and the school years individually. The sample’s normality was not identified, using the Kolmogorov-Smirnov statistical test (*p* > .05), but homogeneity, using the Levene statistical test (*p* > .05). Despite the latter data being favorable for the use of parametric statistics, the first was not adequate; thus, the inferential statistical analyses adopted were of the non-parametric type. The level of significance adopted was *p* ≤ .05. For all analyses, the IBM Statistical Package for Social Sciences (SPSS) 26.0® program was used for Windows®, according to the following description:

- For comparing three or more groups with numerical data, the Kruskal-Wallis test and the Dunn post hoc test were used together. As a measure of effect size, epsilon square (ε^2^) was used, whose reference values were as follows: between 0 < .01 as insignificant; .01 < .04 as weak; .04 < .16 as moderate; .16 < .36 as relatively strong; .36 < .64 as strong; and .64 < 1 as very strong; the Mann-Whitney test was used to compare two independent groups. Cohen’s *d* was used as the effect size. The reference values were .2 as a small effect, .5 as a moderate effect, and .8 great effect
- For comparison between independent groups that had nominal data, the chi-square was used (for expected values < 5, Fisher’s exact test was used). The effect size was verified using Cramer’s V, whose reference values were <.1 as a small effect, .1 to .5 as a medium effect, and > .5 as a great effect
- For correlation between the scores of the instruments, the Spearman rank correlation coefficient (rs) was used; the reference values adopted for such correlations were as follows: weak correlation, values between .00 and .30; moderate correlation, values between .30 and .70; and strong correlation, values between .70 and 1. As a measure of the effect size for this analysis, the coefficient of determination (*r*^2^) was used, whose reference values were .01 for a small effect, .09 for a medium effect, and .25 for a significant effect (Cohen, 1988).

## Results

Among the skills assessed, the first skill that can be cited is creativity, assessed through the TCFI. In Table 1, it is possible to observe the descriptive statistics and the comparison between school years for the raw values of each of its factors, for the general raw total, and the total in percentiles. It is observed that in all factors and in the general totals raw and percentile of the TCFI, the performances did not present statistically significant (*p*) values. However, the effect size revealed a moderate difference in factor 1.

**Table 1:** Descriptive statistics and comparison between school years in administered tests

Variable	1st year M (SD)	1st year Min-Max (Mode)	2nd year M (SD)	2nd year Min-Max (Mode)	3rd year M (SD)	3rd year Min-Max (Mode)	Kruskal-Wallis	Dunn	ε^**2**^
	**Brazilian Test of Children’s Figural Creativity**								
**Factor 1 (enrichment of ideas)**	9.30 (11.15)	0–40 (0)	9.80 (5.02)	1–21 (12)	11.26 (6.89)	2–29 (5.0b)	5.29	1 = 2; 1 = 3; 2 = 3	.07
**Factor 2 (emotivity)**	3.34 (4.35)	0–15 (0)	2.65 (3.21)	0–17 (2)	3.53 (3.77)	0–17 (0)	.818	1 = 2; 1 = 3; 2 = 3	.011
**Factor 3 (creative preparation)**	1.86 (2.39)	0–8 (0)	1.08 (1.12)	0–4 (0)	1.85 (1.64)	0–6 (2)	2.42	1 = 2; 1 = 3; 2 = 3	.03
**Factor 4 (cognitive aspects)**	36.21 (11.70)	15–63 (15,0b)	38.65 (15.02)	8–66 (41.0b)	38.23 (12.29)	19–59 (42)	.798	1 = 2; 1 = 3; 2 = 3	.010
**Raw total**	50.69 (20.97)	16–96 (41.0b)	52.57 (20.24)	12–101 (55.0b)	54.88 (17.76)	27–94 (48.0)	.600	1 = 2; 1 = 3; 2 = 3	.008
**Total percentile**	-	-	34.26 (26.67)	3–87 (3)	35.07 (21.17)	3–71 (40)	326.50ª	2 = 3^a^	.03^b^
	**Abbreviated Wechsler Scale of Intelligence**								
**Vocabulary**	11.08 (5.12)	6–28 (6)	12.76 (4.61)	5–25 (14)	14.88 (4.43)	6–22 (11.0b)	9.72*	1 = 2; 1 < 3; 2 = 3	.13
**Cubes**	5.56 (2.08)	2–11 (6)	6.88 (2.37)	2–15 (6)	9.07 (4.94)	3–20 (6)	7.72*	1 = 2; 1 < 3; 2 = 3	.10
**Similarities**	7.56 (5.12)	1–24 (9.0b)	9.73 (5.32)	2–18 (3.0b)	12.42 (6.86)	3–26 (4)	7.79*	1 = 2; 1 < 3; 2 = 3	.10
**Matrix reasoning**	11.08 (4.45)	5–21 (9.0b)	12.65 (5.09)	5–24 (9.0b)	14.57 (5.90)	4–24 (12.0b)	5.03	1 = 2; 1 = 3; 2 = 3	.06
**Raw total**	35.30 (11.72)	66–108 (73.0b)	42.03 (10.47)	68–96 (72.0b)	50.96 (17.5)	55–96 (66.0b)	13.05*	1 = 2; 1 <3; 2 = 3	.17
**Verbal IQ**	77.13 (12.39)	58–118 (67)	76.07 (10.31)	60–99 (60.0b)	73.42 (12.33)	50–98 (58)	.644	1 = 2; 1 = 3; 2 = 3	.008
**IQ execution**	89.47 (9.29)	71–110 (79)	88.65 (9.19)	72–104 (97)	85.65 (11.33)	65–103 (79.0b)	1.26	1 = 2; 1 = 3; 2 = 3	.017
**Total IQ**	80.82 (9.87)	1–70 (4)	79.76 (7.43)	1–39 (3)	76.65 (11.27)	0.1–39 (1)	.987	1 = 2; 1 = 3; 2 = 3	.013
	**Phonological Awareness - Sequential Assessment Tool**								
**Total syllable**	24.60 (6.19)	10–36 (21.0b)	26.38 (6.78)	12–38 (20)	31.34 (5.91)	17–40 (33)	13.33*	1 = 2; 1 < 3; 2 <3	.18
**Total phoneme**	6.80 (4.43)	0–16 (4b)	9.69 (4.91)	0–19 (10b)	13.96 (5.74)	5–24 (8b)	16.00*	1 = 2; 1 <3; 2 = 3	.21
**Raw total**	30.43 (9.69)	10–52 (24.0b)	36.11 (11.04)	14–55 (21.0b)	44.92 (11.24)	22–62 (49)	17.52*	1 = 2; 1 <3; 2 <3	.23
	**Assessment of Isolated Word and Pseudoword Reading**								
**Raw total**	23.40 (17.15)	3–58 (3b)	30.00 (15.94)	7–53 (29)	44.58 (12.69)	11–59 (51)	16.30 *	1 = 2; 1 <3; 2 <3	.22
**Total percentile**	21.62 (21.89)	7–90 (16)	13.00 (8.80)	7–40 (7)	37.00 (32.33)	10–90 (10)	6,211 *	1 = 2; 1 = 3; 2 <3	.08

^a^Mann-Whitney; *test statistics Kruskal-Wallis or Mann-Whitney with *p* > 0.05; ^b^effect size

Data could not be obtained in the total percentile for the first year as there are no standards for this population. However, it is observed that the percentile averages were low for the second and third years, close to 35, with no statistical difference between both groups, which indicates, according to the normative data, that 65% of the population would probably tend to perform better in this skill.

As for intelligence, measured using the WASI test, in Table 1, it is also possible to observe its descriptive statistics and the comparison between school years. It appears that in the analysis of the raw data of the subtests and the raw total of the test, there were statistical differences between the classes, either by the analysis of significance or by the size of the effect. The post hoc identified a difference in performance when comparing the first and third years, with higher performance in the third, for most subtests. In the comparisons between the first and second and second and third years, the performances were similar. The raw performances in the three types of Intellectual Quotient (IQ) obtained from the four WASI subtests were also described and compared.

It is possible to observe that the performance of the classes for the Verbal IQ was similar. The effect size showed a considerable difference for the Execution IQ and the Total IQ, a moderate difference. The post hoc did not identify specific differences between the groups. In general, low averages of total IQ were obtained, and the ratings ranged from lower-middle and borderline.

The third skill analyzed was phonological awareness using the CONFIAS instrument. In Table 1, it is possible to observe the descriptive statistics and the comparison between school years for each of its two parts of syllabic and phonemic awareness and for the grand total of the test. It is observed that there was a difference between the school years in these three values when both the significance values and the effect sizes were analyzed. The post hoc indicated that the 1st and 2nd years achieved similar performances for the Syllable and Total category, with the 3rd year presenting averages greater than both. For phoneme, the 1st and 2nd years showed similar performances, as did the 2nd and 3rd year, with the 3rd higher only when compared to the 1st year.

For reading decoding, assessed by the LPI test, there was a statistical difference between the 3 years, also when the significance and effect size values were analyzed. In addition, the percentiles were also low, ranging from 13 to 37, and statistically different. The post hoc identified that the 1st and second grades had similar performances, and the third year had a better performance than the other classes for the raw total variable while for the total percentile, the only performance difference found was between the 2nd and the 3rd grade. Also, the minimum and maximum values are described in Table 1, as well as the mode of the variables analyzed for all instruments for a better understanding of outliers and the variation in performance between classes.

As for the correlation analyses, these can be seen in Table 2. It was found that the WASI, in the three school years, correlated with most of the TCFI factors when analyzed by effect size as proposed by Cohen (1988), but showed a small effect and with less reliability in these correlations. When analyzing the correlations by significance, the 1st and 2nd grades showed few correlations between WASI and TCFI, with the 3rd grade with the highest number of positive and significant correlations, more reliable.

**Table 2:** Correlations of TCFI with WASI, CONFIAS, and LPI

**First-year**							
		**TCFI - factor 1**	**TCFI - factor 2**	**TCFI - factor 3**	**TCFI - factor 4**	**TCFI - TB**	**TCFI - TP**
**WASI - Vocab.**	*r*_s;_ *r*^*2*^	.396; .156	.555^b^; .308	.441^a^; .194	−.030; .0009	.311; .096	-
**WASI - Cubes**	*r*_s;_ *r*^*2*^	.319; .101	.180; .032	.068; .004	.243; .059	.353; .124	-
**WASI - Similarities.**	*r*_s;_ *r*^*2*^	−.040; .001	.256; .065	.029; .0008	.101; .010	.059; .003	-
**WASI - Mat. Rea.**	*r*_s;_ *r*^*2*^	.217; .047	.179; .032	.218; .047	−.124; .015	.145; .021	-
**WASI - T. Raw**	*r*_s;_ *r*^*2*^	.336; .112	.474^b^; .224	.310; .096	.003; .000009	.314; .098	-
**WASI - Verbal IQ**	*r*_s;_ *r*^*2*^	.305; .093	.550^b^; .302	.242; .058	.096; .009	.284; .080	-
**WASI - QI Exec.**	*r*_s;_ *r*^*2*^	.414^a^; .171	.373; .139	.214; .045	.110; .012	.388; .150	-
**WASI - Total IQ**	*r*_s;_ *r*^*2*^	.505^a^; .255	.635^b^; .403	.382; .145	.185; .034	.526^b^; .276	-
**CONFIAS - Syllable**	*r*_s;_ *r*^*2*^	.457^a^; .208	.199; .039	.515^a^; .265	.088; .007	.365; .133	-
**CONFIAS - Phoneme**	*r*_s;_ *r*^*2*^	−.055; .003	−.212; .044	−.122; .014	−.243; .059	−.230; .052	-
**CONFIAS - T. Raw**	*r*_s;_ *r*^*2*^	.271; .073	−.033; .001	.221; .048	.022; .0004	.155; .024	-
**LPI - T. Raw**	*r*_s;_ *r*^*2*^	.284; .080	.202; .040	.534^a^; .285	−.270; .072	.093; .008	-
**LPI - T. Percentile**	*r*_s;_ *r*^*2*^	.562^a^; .315	.461; .212	.748^b^; .559	−.148; .021	.394; .155	-
**Second-year**							
		**TCFI - factor 1**	**TCFI - factor 2**	**TCFI - factor 3**	**TCFI - factor 4**	**TCFI - TB**	**TCFI - TP**
**WASI - Vocab.**	*r*_s;_ *r*^*2*^	.139; .019	−.257; .066	.005; .00002	.234; .054	.183; .033	.167; .027
**WASI - Cubes**	*r*_s;_ *r*^*2*^	.275; .075	−.017; .0002	−.129; .016	.187; .034	.191; .036	.183; .033
**WASI - Similarities**	*r*_s;_ *r*^*2*^	.345; .119	.408 ^a^; .166	−.058; .003	.412^a^; .169	.448^a^; .200	.413 ^a^; .170
**WASI - Mat. Rea.**	*r*_s;_ *r*^*2*^	.309; .095	.170; .028	−.300; .09	.092; .008	.151; .022	.154; .023
**WASI - T. Raw**	*r*_s;_ *r*^*2*^	.456^a^; .207	.243; .059	−.178; .031	.436^a^; .190	.477^a^; .227	.454 ^a^; .206
**WASI - Verbal IQ**	*r*_s;_ *r*^*2*^	.323; .104	.159; .025	.028; .0007	.372; .138	.367; .134	.332; .110
**WASI - QI Exec.**	*r*_s;_ *r*^*2*^	.344; .118	.168; .028	−.241; .058	.109; .011	.152; .023	.154; .023
**WASI - Total IQ**	*r*_s;_ *r*^*2*^	.366; .133	.278; .077	−.196; .038	.364; .132	.360; .129	.333; .110
**CONFIAS - Syllable**	*r*_s;_ *r*^*2*^	.507^b^; .257	.071; .005	−.273; .074	.431^a^; .185	.511^b^; .261	.457 ^a^; .208
**CONFIAS - Phoneme**	*r*_s;_ *r*^*2*^	.185; .034	.157; .024	−.411^a^; .168	.140; .019	.210; .044	.150; .022
**CONFIAS - T. Raw**	*r*_s;_ *r*^*2*^	.382; .145	.97; .940	−.329; .108	.280; .078	.367; .134	.303; .091
**LPI - T. Raw**	*r*_s;_ *r*^*2*^	.218; .047	.244; .059	−.430^a^; .184	.190; .036	.242; .058	.197; .038
**LPI - T. Percentile**	*r*_s;_ *r*^*2*^	−.422; .178	−.512; .262	−.391; .152	−.210; .044	−.332; .110	−.339; .114
**Third-year**							
		**TCFI - factor 1**	**TCFI - factor 2**	**TCFI - factor 3**	**TCFI - factor 4**	**TCFI - TB**	**TCFI - TP**
**WASI - Vocab.**	*r*_s;_ *r*^*2*^	.570 ^b^; .324	.327; .106	.475 ^a^; .225	.209; .043	.499 ^b^; .249	.442 ^a^; .195
**WASI - Cubes**	*r*_s;_ *r*^*2*^	.339; .114	.422 ^a^; .178	.288; .082	.468 ^a^; .219	.563 ^b^; .316	.584 ^a^; .341
**WASI - Similarities**	*r*_s;_ *r*^*2*^	.507 ^b^; .257	.331; .109	.533 ^b^; .284	.104; .010	.405 ^a^; .164	.382; .145
**WASI - Mat. Rea.**	*r*_s;_ *r*^*2*^	.459 ^a^; .210	.260; .067	.184; .033	.194; .037	.337; .113	.335; .112
**WASI - T. Raw**	*r*_s;_ *r*^*2*^	−.049; .002	.133; .017	−.016; .0002	−.350; .122	−.331; .109	−.323; .104
**WASI - Verbal IQ**	*r*_s;_ *r*^*2*^	.480 ^a^; .230	.307; .094	.499 ^b^; .249	.102; .010	.400 ^a^; .16	.358; .128
**WASI - QI Exec.**	*r*_s;_ *r*^*2*^	.350; .122	.285; .081	.141; .019	.277; .076	.368; .135	.375; .140
**WASI - Total IQ**	*r*_s;_ *r*^*2*^	.500 ^b^; .25	.390 ^a^; .152	.410 ^a^; .168	.252; .063	.484 ^a^; .234	.468 ^a^; .219
**CONFIAS - Syllable**	*r*_s;_ *r*^*2*^	.748 ^b^; .559	.429 ^a^; .184	.444 ^a^; .197	.272; .073	.548 ^b^; .300	.553 ^a^; .305
**CONFIAS - Phoneme**	*r*_s;_ *r*^*2*^	.666 ^b^; .443	.504 ^b^; .254	.484 ^a^; .234	.250; .062	.534 ^b^; .285	.511 ^a^; .261
**CONFIAS - T. Raw**	*r*_s;_ *r*^*2*^	.774 ^b^; .599	.513 ^b^; .263	.448 ^a^; .200	.309; .095	.599 ^b^; .358	.511 ^b^; .261
**LPI - T. Raw**	*r*_s;_ *r*^*2*^	.647 ^b^; .418	.467 ^a^; .218	.579 ^b^; .335	.324; .104	.616 ^b^; .379	.557 ^b^; .310
**LPI - T. Percentile**	*r*_s;_ *r*^*2*^	.246; .060	.292; .085	.427; .182	.275; .075	.476; .226	.405; .164

*r*_*s*_, Spearman’s correlation coefficient; ^a^ significant value; ^b^ very significant value; *r*^2^, correlation squared; *TB*, raw total; *TP*, total percentile/IQ

Interestingly, the same happened between the TCFI and the reading tests. When the CONFIAS test was correlated with the TCFI, there was a year-on-year increase in the value of the correlations (between the different creative factors and the phonological awareness subtests, as well as between the totals) when analyzing the effect size (Cohen, 1988), however, with an effect weak. As for the analysis of correlations by significance, only in the 3rd grade are there in fact positive and significant correlations. Most were moderate until reaching some values classified as strong. A similar profile was observed between the TCFI and the LPI.

## Discussion

The present study aimed to characterize creativity and verify its relationships with intelligence, phonological awareness, and reading decoding in Brazilian children belonging to the mandatory school initials in the country, specifically, from the first to the third year of Elementary School I. In relation to performance in creativity, one of the hypotheses corroborated by the literature is its evolution in children during the schooling process, which would become more creative as they progress and become older due to factors such as identity and personality construction, greater school experience, and better capacity development cognitive and emotional (Claxton et al., 2005; Hansenne & Legrand, 2012; Wu et al., 2005). However, the present study does not confirm this finding, as the TCFI showed a difference in performance in only 1 of its factors between school years.

Two hypotheses can be developed from this result. First, creativity during childhood goes through different developmental variations, with declines especially during elementary school (Urban, 1991). Thus, as this is a sample of children with similar ages and in the literacy period, the creative performance does not show major differences. Characteristics such as learned knowledge, thinking styles, verbal and language skills, types of stimulation, and motivation are also responsible for performance throughout child development for creativity (Wu et al., 2005). Another aspect that may have impacted the similar performance of creative skills were the traditional teaching methods, characteristics of schools in terms of structure, lack of materials, and poor working conditions for teachers due to little investment by the State. In this sense, the environment plays an important role, as it can encourage creativity, as this is where the child spends most of their time. As such, an adequate physical environment, availability of material, outdoor activities, flexible teaching, and use of play will contribute to the natural development of creativity (Davies et al., 2013; Ershadi & Winner, 2020). When comparing traditional schools and schools with alternative education systems, children from the latter system usually perform better in creative skills (Castillo-Vergara et al., 2018; Runco & Charles, 1997).

The effects of these developments, schooling, and age are also expected for intelligence. In other words, the longer formal education, the higher the scores on intelligence tests, possibly benefiting the intellectual aspects, especially those assessed on IQ tests, which have tasks strongly related to the school context (Cliffordson & Gustafsson, 2008; Ritchie & Tucker-Drob, 2018; Roth et al., 2015). In the present investigation, gains were observed in the raw scores as the school year increased, being more statistically evident between the 1st and third grades in 3 of the 4 intelligence subtests and in the grand total.

The school, in addition to being the place where children acquire new information and concepts, is also responsible for the evolution of intellectual skills, with intelligence being a measure related to performance in educational practices (Ribeiro & Freitas, 2018). The 3rd grade has a relative advantage over other school years, due to the active participation in school activities through the subjects of Portuguese, science, and mathematics, as well as greater participation in school subjects and activities, a hypothesis strongly raised in the literature due to the g factor of intelligence have moderate to large relationships with educational achievement over school years (Calvin et al., 2010).

It is also added that as cognitive development occurs up to a maturational level, the effect of children’s age decreases and the influence of education becomes more relevant to intelligence, which would partly explain the non-progression of the skill between 1st and 2nd grades, being an initial period of schooling and general intellectual aspects (Wang et al., 2015). Therefore, the biggest challenge for scholars in the field is to be able to understand the individual factors inherent to child neurodevelopment during this trajectory, considering the various factors that affect the ability, in an attempt to identify which aspects drive these changes in intellectual development (Gomes & Golino, 2012).

For performance in reading skills, primarily for phonological awareness, the 3rd grade had better overall performance. At this stage of development, it is possible to observe the effect of the level of schooling consistently on the development of phonological awareness, as the ability as a metalinguistic competence is consolidated as the child progresses in school with exposure to formal content and advancement in literacy; thus, older children would perform better for more extended teaching (Cardoso et al., 2013; Leite et al., 2018). However, no significant differences were found between the 1st and the 2nd year, which is possibly justified by the not yet proficient development of the skill in children in the literacy process, especially for those with difficulties in reading and learning. Thus, it would be expected to the absence of skill evolution in the early years (1st and 2nd grades) (Cardoso et al., 2013; Santos et al., 2017), as the children in this sample had difficulty performing the phonological awareness activities.

The difficulties observed in this skill may have had an effect on the performance of decoding tasks by the LPI, since an adequate development of phonological skills is necessary for word recognition (decoding), which was evidenced by low percentiles among the 3 classes in reading the words. As an explanation for these performances, children from participating schools did not learn the sounds of letters and there was no stimulation of predictive reading skills. Thus, the methodological practices employed might not be sufficient for such development. Another hypothesis refers to the socioeconomic context and family characteristics of children. Investigations of low-income Latin American children indicate slower reading acquisition due to socioeconomic variables, lack of stimulation at home, health, nutrition, and parental education level that may play an important role in primary reading skills (Diuk & Ferroni, 2013; González Seijas et al., 2017; Ozernov-Palchik et al., 2019).

When comparing the performance between classes for decoding by raw scores, the results are similar to other findings with children in schooling, indicating the better performance of the most advanced class in school (Leite et al., 2018; Santos et al., 2017). This result is also similar for phonological awareness, which indicated the best performance in the 3rd year when compared to the 1st and 2nd. Possibly, there is again, as a primary reading skill, the hypothesis of the effect of schooling and longer teaching time, which confirm that word recognition in children aged 6 to 10 years is better in older children due to the maturation of cognitive components (Seabra & Dias, 2012).

From the above, another essential variable to analyze is the standardized scores obtained in the tests. It was possible to measure them in the TCFI and LPI tests by the percentile and the WASI test by the IQ. In the first, used to assess creativity, these data were obtained for the 2nd and third years, with scores below the average and no difference between them. At LPI, there was also a normative performance below the average in the three school years, but with gains in the 3rd. In the IQ, decreased averages were also obtained in the 3 years, with differences between them, but so subtle that the post hoc did not identify them.

These data become worrying as they lead to identifying cognitive and school problems in the investigated population. Among the hypotheses elaborated that could explain this result, we have that (a) the tests used do not present specific standards for the region of northeastern Brazil and (b) the context of vulnerability of the children in the sample. Regarding the first, the Federal Council of Psychology (Conselho Federal de Psicologia – CFP, 2018) recognizes that to have optimal qualities in a Brazilian test, it is necessary to investigate it, mainly normative, in the different regions of the country. Because Brazil is a continental country, there are evident differences in cultural, linguistic, and socioeconomic variables, which must be ensured mainly in the correction of an instrument, as they interfere with its score. When analyzing the manuals of all these instruments used, data from the Northeast were not identified. However, the instruments that exist in the country with psychometric qualities are rare and scarce, and these were chosen for the research because they have one of the most complete. However, it is likely that, specifically, your standards may then have provided low scores in the sample.

As for the second hypothesis, even though the social context variable was not investigated in the study, it is essential to highlight the profile presented by the sample. The students came from municipal public schools with low IDEB (Basic Education Development Index). The IDEB as an indicator of the quality of education is directly related to the socioeconomic level of schools and students (Mello & Bertagna, 2016; Panassol, 2020; Souza et al., 2019) and the performance on this indicator goes beyond of individual student results. Currently, there is strong evidence that the low performance in this indicator is related to the physical conditions of schools and the investment in pedagogical and technological material goods, library use, access to books, filtered water, as well as the conditions of the family environment of children, consumer goods, quality of life and food, active participation of family members, and literate parents, among others (Fillipin et al., 2020; Lourenço et al., 2017).

Thus, better study opportunities with access to various teaching materials and educated parents would be important factors to help children at school, in addition to favoring the proper development of cognitive and emotional skills needed at this stage of development, consequently improving IDEB indexes (Mello & Bertagna, 2016). These findings further support the view that the quality of education, school performance, and these cognitive skills will also be influenced by social variables associated with poverty and unequal access to education (Jacob et al., 2020).

As for the relationship between creativity and intelligence, there were significant correlations in all classes by significance or effect size. But, as the correlations by significance proved to be more reliable, it can be understood that in the 3rd class the relationships between skills are stronger. Even though the correlations by significance in the initial years (1st and 2nd) are few, they grow year by year, and the correlations by effect size are weak, this data points to an interesting finding about the relationship between creativity and intelligence. The hypothesis elaborated here for this result is that these skills can be related after a certain level of schooling. Kim (2006) demonstrated that groups with a higher level of schooling, at the end of elementary school, high school, and higher education, have intelligence scores more easily associated with creativity scores. In younger children, it would be weaker, probably due to the educational influence and cognitive skills still in development.

Similar results are also found in national and international studies with elementary school children (Gonçalves & Fleith, 2011; Krumm et al., 2018). The greatest contribution of these works is the predictive factor that fluid intelligence exerts on creativity, especially in the skills of fluency, originality, creative responses, and development of analytical strategies in divergent thinking tasks (Batey et al., 2010; Silvia, 2015). However, the literature points out that there is no general consensus on this association, as, in contrast, crystallized intelligence is also a necessary factor in this relationship, as a competence to use the knowledge learned to solve and solve problems. In this way, it plays an important role for divergent thinking and favors creativity with access to acquired information (Batey et al., 2009; Cho et al., 2010).

Conversely, for the present study sample, both the verbal subtests representing crystallized intelligence measures and the execution subtests representing WASI fluid intelligence measures correlated significantly with the factors of creativity. Possibly, individuals use aspects of acquired knowledge (crystallized intelligence) and ideas from fluid reasoning competence (fluid intelligence) in daily routine as there is school influence and advancing age (Batey et al., 2009).

By the CHC theory (McGill & Dombrowski, 2019), intelligence can be divided between 18 factors, including fluid (Gf) and crystallized (Gc) intelligence. There is also evidence of moderate and strong relationships between creativity and two other factors, broad retrieval ability (Gr) and manipulation of spatial stimuli (Gv) (Frith et al., 2021). These factors are also evaluated by WASI and its subtests and it is possible to find significant correlations in 3rd grade. Possibly, the Gr factor representing the ability to retrieve important concepts from long-term memory can contribute to the production and rescue of creative ideas (Benedek et al., 2014; Silvia et al., 2013). The Gv factor, which represents the manipulation and reorganization of three-dimensional objects, can contribute to creative skills focused on mathematics, science, and technology (Wai et al., 2009).

As for the most discussed hypotheses about the relationship of abilities, high intelligence is not necessary for high creativity or even that creativity is essential for intelligent behavior to develop (Sternberg & O'Hara, 1999). The findings of the present study do not confirm these possibilities, as none of the groups showed good performances in intelligence or creativity and, even so, there were still significant correlations.

In addition, another intensely discussed hypothesis is that from a specific IQ threshold, creativity, and intelligence would be related. As observed in a study by Sligh et al. (2005), strong correlations between fluid and crystallized intelligence with creativity were found between participants of medium and high IQs. Likewise, Cho et al. (2010) observed relationships between skills based on average IQ performances. In contrast, in the results found for the present sample, even with IQs and creativity scores below average, significant and moderate correlations were still found. Possibly students with low IQ scores can also be creative and produce rare ideas, even if these are not useful and original (Kim, 2006).

Another interesting finding is between the factors of TCFI and WASI. When analyzing correlations by significance, the factors relate differently to the WASI for each school year. The 3rd grade is the class in which the most significant correlations occurred, with factor 1 (enrichment of ideas) and factor 3 (creative preparation) showing more consistent relationships with the aspects of intelligence. Possibly because factor 1 involves good organizational and planning skills, in addition to the ability to perceive stimuli differently, understanding the problem in a global way and was shown to be correlated with the matrix reasoning subtests, similarities, in addition to verbal IQ, in that in these activities abstract thinking is also requested. Factor 3, on the other hand, is involved in the analysis of response possibilities and choice of the most appropriate solution, an aspect that would explain the correlations found between this factor with the verbal IQ and the verbal subtests of vocabulary and similarities. Interestingly, due to effect size correlations, despite having a small impact, factor 2 (emotionality) correlated with almost all subtests and IQs from WASI, one of the most encouraged findings in the literature on the importance of emotional variables in the development of creativity (Lubart, 2007).

As for the 1st grade classes, factor 1 (by effect size, weak) and factor 2 (by significance, moderate and high) correlated to a greater degree with the verbal activities of the WASI. These factors are responsible, respectively, for good organizational, planning skills, emotional skills, and persistence, which would demonstrate that these skills can be involved in verbal skills. For the 2nd year, most of the correlations found are between factors 1 and 4, however, only when the effect size is analyzed, therefore, with correlations that are not especially reliable, being also found negative correlations between the factors of the TCFI and to WASI, which could indicate that the relationship of these specific creativity skills with intelligence in the 2nd year would be more complex.

For the possible relationships between creativity and reading skills, first, as observed in the characterization of both, the children showed low performance in reading and vocabulary decoding, with significant difficulty in carrying out the phonological awareness tasks, without proficient reading by parts of the classes. Poor reading acquisition could impact on the analyzed relationships; as observed by the 1st and 2nd years, they showed few correlations by significance and weak correlations by effect size between creativity, phonological awareness, and reader decoding; differently, the 3rd year showed correlations between moderate to strong. Considering that no previous studies were found, some hypotheses can be developed for the results found.

Once again, for the findings of the initial years (1st and 2nd grades), the issue of little developmental maturation may have influenced the weak or few correlations found (Roth et al., 2015), which would explain the 3rd year having presented more significant and reliable correlations. In this sense, for this relationship found, these findings only reinforce the theory of “Creative Reading” which defends creativity as a basis for quality reading. In this theory, reading comprehension depends on learning new concepts and this action is a function of creativity (Popov, 1992). This understanding could explain the significant correlations found between creativity and phonological awareness in the 3rd grade class, through the underlying cognitive skills that commonly involve them, as there is evidence that metacognition can moderate creativity (Preiss et al., 2019). Correlations of specific creativity skills can be observed, in factors 1 (enrichment of ideas), 2 (emotionality), and 3 (creative preparation), positively correlating with all factors of CONFIAS, at syllabic, phonemic, and total levels. From the definition of creativity by Torrance (1974), used in this research and also in the TCFI, creativity demands actions of identification, production, creation, and choice of the best and adequate answer. Likewise, CONFIAS involves actions of synthesis, segmentation, identification, production, and exclusion of stimuli. Torrance also built a test of verbal creativity (Wechsler, 2004), which unfortunately is not validated in Brazil for children and involves these same principles.

The relationships between creativity and reader decoding assessed respectively through the TCFI and the LPI showed similar results to CONFIAS; when analyzing the correlations by significance, 1st and 2nd grades again showed few correlations compared to the 3rd grade; and despite the fact that correlations are shown in greater numbers when analyzing the effect size, this is small. In addition, negative correlations are also observed in the initial classes, a finding that suggests that between creativity and predictive reading skills such as decoding, this relationship could not happen. As previously proposed, it is believed that these abilities could be related only from a specific level of cognitive maturation.

And as indicated, the skills were more strongly related in the 3rd year. No studies were found on such relationships; however, they were found on creativity and reading skills related to decoding, such as reading comprehension and speed in the respective studies by Anderson and Gipe (1983), Sturgell (2008), Wang (2012), and Saeed et al. (2013). These authors concluded that the participants who had better reading habits in their routine and higher scores for text comprehension and reading speed were those who demonstrated the best performance in creativity. Using this parameter, the 3rd grade, the class with the greatest educational experience, had better performance in the raw total of the LPI, in most of the phonological awareness activities and in the vocabulary skill in WASI, aspects that could explain greater correlations in this class, despite the performance in creativity has remained constant among the 3 classes. This hypothesis can also be reinforced by the correlations found between factors 1, 2, and 3 of the TCFI with the LPI scores. The factors mentioned are involved in different stages of creative production, from the production and organization of ideas, the use of emotional control, and persistence to the process of choosing the most appropriate idea/stimulus to put into practice (Nakano et al., 2011).

This was probably due to greater time spent on activities aimed at reading skills developed linguistic and cognitive skills that are also involved in creativity, such as elaboration, in the sense of creating details that enrich ideas; originality, the ability to develop rare ideas; and especially the fluency skill, in which the student can produce a diversity of ideas in a given time. Skills that can be achieved through reading and are determinants for creative performance. It also adds to this relationship the presence of skills such as freedom of expression, curiosity, and problem-solving skills (Wang, 2012). As seen in qualitative evidence, creative characteristics associated with problem solving, freedom of expression, behavior monitoring, curiosity, and playfulness, among others, can moderate performance in reading activities (Franco & Balça, 2018; Muniz & Martínez, 2015). If encouraged and employed in school curricula, creativity could play an important role in voluntary involvement and involvement in learning such skills.

Added to this perspective, creativity in reading would enable the development of cognitive skills, language, imagination, freedom of expression and reading comprehension, the development of cognitive and metacognitive, language skills, and the development of more qualitative skills such as imagination, playfulness, and freedom of expression (Jończyk et al., 2020; Preiss et al., 2019). In this sense, there would be a “facilitation” between reading and creativity, in which skills would be related through shared biological/cognitive processes and also through the effects of increased vocabulary capacity in the act of reading (Ritchie et al., 2013).

Overall, creativity was shown to be related to reading skills for all classes, in agreement with empirical studies that indicate the relationship of skills; however, as discussed, the strength of this relationship is diverse, proving to be weak (Bart et al., 2020), data that reinforce the weakness of these constructs and the absence of an adequate methodology for assessment, which is an important finding for future studies that can deepen this investigation.

As with creativity and intelligence, it is possible that reading skills are related to the first and will increase as schooling and age develop as a result of the integration between them that is socially demanded. Such integration between school and cognitive skills can be better understood even by the CHC theory of intelligence, which proposes empirically and theoretically that a higher hierarchy of skills would be explained by a common factor, the g factor (McGill & Dombrowski, 2019).

## Conclusions and limitations

As this is an exploratory study, it is concluded that the findings regarding the relationship between creativity and other skills were promising, considering that significant correlations were found for the classes. However, the study had some limitations. Firstly, as the statistical analysis pointed out, the effect size for the correlations found was small, so we must observe the relationships found between creativity with intelligence and reading skills with caution, especially with regard to the generalization of the results. A relatively small sample was used with very high performance variability across the sample, an issue that may have had a considerable impact on the relationships found between the skills. Investigations with wider samples could help to better understand how these skills are truly related in addition to the influence of the social context associated with poverty. It is also indicated for future studies, the control of variables such as intelligence, personality, and social context (socioeconomic level, family characteristics) that were not controlled in the present study, as they are important aspects in the development of creative skills. Another limitation was the use of a creativity instrument that did not present data for the 1st class, the only instrument validated in the country, that care should also be taken to formulate national psychometric tests involving this region, at least in its standards.

As observed in the present study, the development and relationship of creativity with other skills is still complex; however, the important relationships found between creativity with intelligence and reading, especially in the 3rd year class, demonstrate the importance of fostering creative skills in the classroom beyond issues such as intelligence or reading, but in the academic curriculum itself, since creativity can favor the development of more inclusive practices that are adaptable to the diversity of students during elementary school. From the above, education professionals who want to encourage collaborative learning that respects the differences of each child, especially those arising from vulnerable contexts, must provide spaces and methodologies that involve creativity. The school is still the basic place where a fundamental part of the child’s development and the schooling process takes place, so it is natural to think that the educational system can prepare students for current times and for the future. These aspects also demand an individual who knows how to deal with self-confidence, leadership, persistence to continue, and courage to take risks. Another important finding that translates into necessary practical implications in schools, especially in public and with a high level of poverty, is the development of specialized educational interventions for primary reading skills, since limiting performances were found for such competencies and may have an unfavorable impact on acquisition and development of reading in the following years.

We suggest the continuation of new studies that may contribute to the investigation of creativity, intelligence, and the development of reading in the early years of literacy to better understand these skills in the educational context and especially in the northeastern region of Brazil, the reality of children still little investigated. Based on the characterization of the skills explored in the study, future practices can be designed and teacher assistance can be done for better instruction, with a view to accessibility to a learning culture that thinks of the collective. Since the stimulation of creative ability provides benefits in the context of education for the typical population and, especially, for children who are at risk for the development of learning difficulties.

## Data Availability

The data sets used and/or analyzed during the current study are available from the corresponding author upon reasonable request.

## Abbreviations

TCFI: Test of Children’s Figural Creativity
WASI: Wechsler Abbreviated Scale of Intelligence
VIQ: Verbal Intellectual Quotient
IEQ: Intellectual Execution Quotient
IQ: Intellectual Quotient
CONFIAS: Phonological Awareness - Sequential Assessment Tool
LPI: Reading Assessment of Words and Pseudowords Isolated
